# Taste Cells of the Type III Employ CASR to Maintain Steady Serotonin Exocytosis at Variable Ca^2+^ in the Extracellular Medium

**DOI:** 10.3390/cells11081369

**Published:** 2022-04-18

**Authors:** Aleksandr P. Cherkashin, Olga A. Rogachevskaja, Natalia V. Kabanova, Polina D. Kotova, Marina F. Bystrova, Stanislav S. Kolesnikov

**Affiliations:** Pushchino Scientific Center for Biological Research of the Russian Academy of Sciences, Institute of Cell Biophysics of the Russian Academy of Sciences, Pushchino 142290, Russia; leva-trockiy@yandex.ru (A.P.C.); o.rogachevskaja@gmail.com (O.A.R.); kabanovatata@mail.ru (N.V.K.); polinakotova88@gmail.com (P.D.K.); marinabystrova@rambler.ru (M.F.B.)

**Keywords:** taste cells, voltage-gated Ca^2+^ channels, serotonin secretion, serotonin biosensor, extracellular Ca^2+^-sensing receptor

## Abstract

Type III taste cells are the only taste bud cells which express voltage-gated (VG) Ca^2+^ channels and employ Ca^2+^-dependent exocytosis to release neurotransmitters, particularly serotonin. The taste bud is a tightly packed cell population, wherein extracellular Ca^2+^ is expected to fluctuate markedly due to the electrical activity of taste cells. It is currently unclear whether the Ca^2+^ entry-driven synapse in type III cells could be reliable enough at unsteady extracellular Ca^2^. Here we assayed depolarization-induced Ca^2+^ signals and associated serotonin release in isolated type III cells at varied extracellular Ca^2+^. It turned out that the same depolarizing stimulus elicited invariant Ca^2+^ signals in type III cells irrespective of bath Ca^2+^ varied within 0.5–5 mM. The serotonin release from type III cells was assayed with the biosensor approach by using HEK-293 cells co-expressing the recombinant 5-HT4 receptor and genetically encoded cAMP sensor Pink Flamindo. Consistently with the weak Ca^2+^ dependence of intracellular Ca^2+^ transients produced by VG Ca^2+^ entry, depolarization-triggered serotonin secretion varied negligibly with bath Ca^2+^. The evidence implicated the extracellular Ca^2+^-sensing receptor in mediating the negative feedback mechanism that regulates VG Ca^2+^ entry and levels off serotonin release in type III cells at deviating Ca^2+^ in the extracellular medium.

## 1. Introduction

The mammalian taste bud represents a heterogeneous population of different cells, including type I–type III taste cells and precursor basal (type IV) cells [1,2]. The existing evidence implicates type III cells in mediating sour taste [3] as well as taste bud responsivity to carbonation [4] and salts at high concentrations [5,6]. Type III cells are electrically excitable [7,8] and generate action potentials (APs) in response to taste stimulation [9,10]. In the taste bud, only type III cells employ classical chemical synapses [11] and express voltage-gated (VG) Ca^2+^ channels [7,8,12,13]. The transcriptome analysis and functional assay suggest that type III cells employ diverse VG Ca^2+^ channels, including L-type (CaV1.2, CaV1.3), N-type (Cav2.2), and P/Q type (Cav 2.1) [13,14]. To all appearances, the main function of VG Ca^2+^ channels in type III cells is to mediate AP-driven Ca^2+^ entry that stimulates exocytosis of neurotransmitters, including serotonin and presumably ATP [15,16].

In the taste bud, cells are packed very densely [11,17]. The taste bud morphology suggests that the extracellular space is a restricted compartment of submicron size, so that by volume, it is much smaller, by 2–3 orders, than the cell cytosol. It, therefore, could be expected that in the stimulated taste bud, extracellular ions, especially Ca^2+^, deviate markedly from stationary levels, as the electrical activity of taste cells redistributes ions between the spacious cell cytosol and the small extracellular space. In the case of Ca^2+^ homeostasis, one more significant factor is the high buffering capacity of the cell cytoplasm, which is commonly characterized by the ratio β = Δ(bound Ca^2+^)/Δ(free Ca^2+^). Among *N* Ca^2+^ ions translocated from the extracellular medium into the cytosol, most should be bound, and only *N*/(1 + β) ions remain free. With the effective extracellular and intracellular volumes V_out_ and V_in_, respectively, the translocation of *N* Ca^2+^ ions into the cytosol would decrease extracellular Ca^2+^ concentration by ΔC_out_ = *N*/V_out_, while free intracellular Ca^2+^ would be increased by ΔC_in_ = *N*/((1 + β) V_in_). Given that β > 100 in apparently all assayed cells [18] and that V_in_/V_out_ > 10^2^, ΔC_out_/ΔC_in_ =(1 + β) V_in_/V_out_ > 10^4^. This estimate suggests that the 100 nM increase in cytosolic Ca^2+^ mediated by Ca^2+^ entry into a taste cell should be accompanied by the local drop in extracellular Ca^2+^ of about 1 mM. If so, the question arises of whether the Ca^2+^ entry-driven synapse in type III cells could be reliable enough while extracellular Ca^2+^ is unsteady to a high extent. Here, we addressed this issue by assaying depolarization-elicited Ca^2+^ signals in isolated type III taste cells and associated serotonin release at varied Ca^2+^ in the bath.

## 2. Matherials and Methods

### 2.1. Isolation of Taste Buds and Cells

All experimental protocols were in accordance with local regulatory bodies and the European Communities Council Directive (2010/63/EU) and approved by the Commission on Biosafety and Bioethics (Institute of Cell Biophysics—Pushchino Scientific Center for Biological Research of the Russian Academy of Sciences, Permission no. 4/062020 (12 June 2020). Taste cells and taste buds were isolated largely from mouse circumvallate papillae and sometimes from foliate papillae as described previously [19]. Briefly, an isolated tongue was injected between the epithelium and muscle layers with 1.0 mg/mL collagenase B, 1.8 mg/mL dispase II, 0.4 mg/mL elastase, and 0.5 mg/mL trypsin inhibitor (all from Sigma-Aldrich, St. Louis, MO, USA) dissolved in a solution (mM): 120 NaCl, 20 KCl, 1 MgCl_2_, 1 CaCl_2_, 10 glucose, 20 HEPES-NaOH (pH 7.8). Once injected, a tongue was incubated in the oxygenated low-Ca^2+^ solution (in mM): 130 NaCl, 10 KCl, 0.7 CaCl_2_, 1.1 MgCl_2_, 1 EGTA, 1 EDTA (calculated free Ca^2+^ was 115 nM), 10 HEPES-NaOH (pH 7.4), 10 glucose) for 30–35 min. After the treatment, a lingual epithelium was peeled off from the underlying muscle, pinned serosal side up in a dish covered with Sylgard resin, and then it was incubated in the Ca-free solution for 10–30 min. The isolated epithelium was kept at room temperature in a solution (in mM: 130 NaCl, 5 KCl, 1 MgCl_2_, 2 CaCl_2_, 10 HEPES-NaOH (pH 7.4), 10 glucose, 5 Na-pyruvate) for 4–6 h. To obtain individual taste buds, those were removed from a CV papilla by gentle suction using a fire-polished pipette with an opening of 80–100 μm. Taste cells were isolated by using suction pipettes with tips of 50–70 μm. The obtained cellular material was then expelled into an electrophysiological or photometric chamber.

### 2.2. Electrophysiology

Ion currents were recorded, filtered, and analyzed using an Axopatch 200 B amplifier, Digidata 1440A, and pClamp 10 software (all from Molecular Devices, LLC., San Jose, CA, USA). External solutions were delivered by a gravity-driven perfusion system at a rate of 0.1 mL/s. Cells were typically polarized by serial voltage pulses of appropriate duration at a 10 mV step. Generally, the perforated patch approach was used, and recording pipettes were filled with (mM): 140 CsCl, 1 MgCl_2_, 0.1 EGTA, 10 HEPES-CsOH (pH 7.2), 400 µg/mL amphotericin B. The basic bath solution included (mM): 140 NaCl, 5 KCl, 1 MgCl_2_, 2 CaCl_2_, 10 HEPES-NaOH (pH 7.4), 10 glucose. When needed, 140 mM NaCl + 5 mM KCl was replaced with 75 mM NaCl + 70 mM KCl and/or CaCl_2_ was added at a concentration of 0.5, 1, or 5 mM. The recording chamber of nearly 75 μL has been described previously [20].

### 2.3. Ca^2+^ Imaging 

Isolated cells were plated onto a photometric chamber of nearly 150 μL volume. The last was a disposable coverslip (Menzel-Glaser, Waltham, MA, USA) with an attached ellipsoidal resin wall. The chamber bottom was coated with Cell-Tak (Corning, NY, USA) ensuring sufficient cell adhesion. Attached cells were loaded with the Ca^2+^ dye Fluo-8 at room temperature (23–25 °C) by adding Fluo-8AM (4 μM) (AAT Bioquest, Sunnyvale, CA, USA) and Pluronic (0.02%) (Molecular Probes, Waltham, MA, USA) to the bath. As being prone to quick rundown, isolated taste cells were loaded for 15 min, rinsed with the bath solution several times, and immediately assayed. HEK-293 cells were loaded for 20 min, rinsed several times, and kept in the bath solution for 30 min prior to recordings.

Experiments were carried out using an inverted fluorescent microscope Axiovert 135 equipped with an objective Plan NeoFluar 20×/0.75 (Carl Zeiss, Inc., Chicago, IL, USA), a digital ECCD camera LucaR (Andor Technology, Belfast, UK), and a hand-made computer-controllable epi-illuminator with a set of light-emitting diodes, particularly allowing for the excitation of Fluo-8 at 480 ± 10 nm. The Fluo-8 emission was collected at 535 ± 25 nm. Serial fluorescent images were captured every second and analyzed using Imaging Workbench 6 software (INDEC BioSystems, Los Altos, CA, USA). Deviations of cytosolic Ca^2+^ from the resting level in individual Fluo-8-loaded cells were quantified by the ratio ΔF/F_0_, where ΔF = F − F_0_, F is the instant intensity of cell fluorescence, F_0_ is the intensity of cell fluorescence obtained in the very beginning of a recording and averaged over a 20 s interval. All chemicals were applied by the complete replacement of the bath solution in a 150 μL photometric chamber for nearly 2 s using a perfusion system driven by gravity.

The used salts, buffers, spermine, and serotonin were purchased from Sigma-Aldrich (St. Louis, MO, USA); NPS-4123 and NSP R-568 were from Tocris Bioscience (Bristol, UK).

### 2.4. Serotonine Release Assay

The serotonin release from taste cells was assayed using the cellular biosensor approach [21]. Cells of the HEK-293 line, which heterologously expressed the metabotropic serotonin receptor 5-HT_4_ coupled to adenylyl cyclase and genetically encoded cAMP sensor Pink Flamindo, served as a serotonin biosensor. The receptor 5-HT_4_ was cloned from the mouse brain (described in more detail in Section SI), while the genetically encoded cAMP sensor Pink Flamindo was a gift from Tetsuya Kitaguchi (Addgene plasmid #102356; http://n2t.net/addgene:102356, accessed on 1 October 2019; RRID:Addgene_102356 [22]).

HEK-293 cells were routinely cultured in Dulbecco’s Modified Eagle’s Medium (DMEM) (Invitrogen, Waltham, MA, USA) containing 10% (*v*/*v*) fetal bovine serum (HyClone, Marlborough, MA, USA), glutamine (1%), and the antibiotic gentamicin (100 μg/mL) (Invitrogen). Cells were grown in 12-well culture plates in a humidified atmosphere (5% CO_2_/95% O_2_) at 37 °C. To induce expression of the 5-HT_4_ receptor and Pink Flamindo sensor, HEK-293 cells were transfected with two appropriate plasmid vectors. Transfected cells were then subjected to selection to obtain cellular monoclones, which stably expressed 5-HT_4_ and Pink Flamindo, thus being responsive to nanomolar serotonin with cAMP transients detectable by Pink Flamindo fluorescence (Section SI). 5-HT_4_/PF cells were cultivated in the presence of 400 μg/mL G-418 + 2 μg/mL puromicin (both from Invitrogen, Waltham, MA, USA).

## 3. Results

### 3.1. Influence of Extracellular Ca^2+^ on VG Ca^2+^ Currents and Related Ca^2+^ Transients

**Figure 1:**
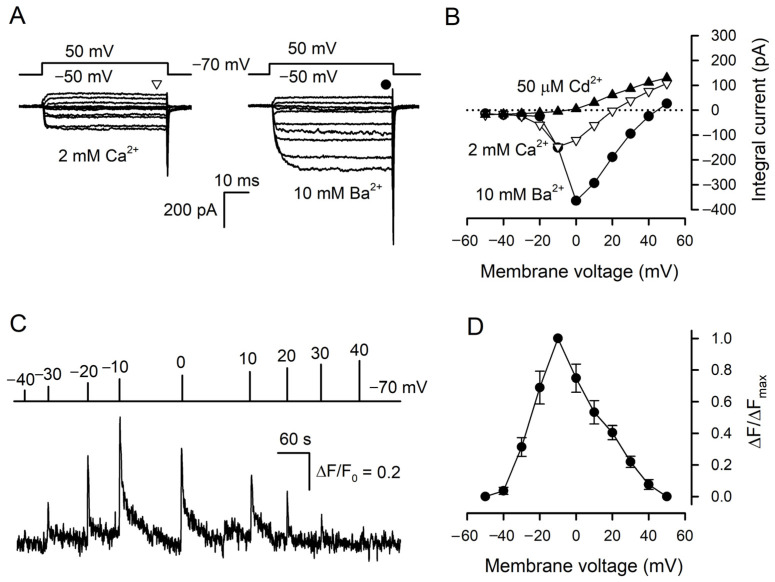
Cytosolic Ca^2+^ transients elicited by VG Ca^2+^ entry in type III cells. (**A**) Representative (*n* = 34) perforated patch recording of integral currents under conditions highlighting VG inward Ca^2+^ or Ba^2+^ currents through VG Ca^2+^ channels with 2 mM Ca^2+^ or 10 mM Ba^2+^ in the bath, respectively. The cell was held at −70 mV and polarized from −50 to 50 mV by 50 ms voltage pulses with 10 mV increment. To compensate recordings for leak currents, the P/4 leak subtraction protocol was used. The patch pipette contained 140 mM CsCl and amphotericin B (400 μg/mL) and the bath solution contained 2 μM TTX. The sustained VG currents were evaluated in the moments indicated by the symbols above the current traces. (**B**). Voltage dependence of the sustained VG Ca^2+^- (∇) or Ba^2+^ (●) currents recorded in control (**A**) and in the presence of 50 μM Cd^2+^ (▲). (**C**) Representative monitoring (*n* = 12) of intracellular Ca^2+^ in a patch-clamped type III cell loaded with Fluo-8. The cell was held at −70 mV and polarized by 100 ms voltage pulses from −40 to 40 mV to stimulate Ca^2+^ entry at the moment indicated by the curve above the Ca^2+^ trace. Recording conditions as in (**A**). (**D**) Averaged Ca^2+^ transient versus membrane voltage. The data are presented as the mean ± S.D. (*n* = 12).

Taste cells were isolated from mouse circumvallate (CV) papillae and examined with the perforated patch-clamp technique and Ca^2+^ imaging. Taste cells of a particular subtype could be reliably identified by a characteristic set of VG currents that they exhibited under pseudophysiological conditions [8,23]. In particular, solely type III taste cells functionally express VG Ca^2+^ channels [7,8,12]. With 140 mM CsCl in the patch pipette that suppressed VG K^+^ currents, type III taste cells became even more authentic electrophysiologically [24], while the inhibition of VG Na^+^ channels with 100 nM TTX allowed for rectifying the activity of VG Ca^2+^ channels more clearly. Under these conditions, nearly 90 type III cells were assayed overall. Usually, these cells exhibited subtle VG Ca^2+^ currents of 30–100 pA at 2 mM bath Ca^2+^ (Figure 1A, left panel), which completely disappeared in the presence of 50 μM Cd^2+^ in the bath (Figure 1B). The substitution of 2 mM Ca^2+^ for 10 mM Ba^2+^ markedly enhanced currents through VG Ca^2+^ channels (Figure 1A, right panel). In designated experiments, taste cells were loaded with Fluo-8, patch clamped, and assayed with Ca^2+^ imaging to examine Ca^2+^ signals initiated by VG Ca^2+^ entry. Overall, we found 12 quite robust type III cells with active Ca^2+^ channels, which allowed for the stable concurrent monitoring of VG Ca^2+^ currents and intracellular Ca^2+^ for a sufficiently long period. Being depolarized by 100 ms voltage pulses of variable magnitude, these cells showed voltage-dependent Ca^2+^ transients (Figure 1C). Consistently with the I–V curve characterizing VG Ca^2+^ currents at 2 mM bath Ca^2+^ (Figure 1B), these depolarization-induced Ca^2+^ signals were maximal at −10 mV (Figure 1D).

Next, we analyzed the dependence of VG Ca^2+^ currents on extracellular Ca^2+^. The value of this current at a particular voltage was calculated as a difference between integral VG currents recorded in the control and in the presence of 50 μM Cd^2+^. Note that Ca^2+^ ions entering through VG Ca^2+^ channels into the cytosol can affect their activity through several negative feedback mechanisms [25]. To prevent the possible influence of this intracellular factor on the dependence of VG Ca^2+^ currents on bath Ca^2+^, taste cells were loaded with the fast Ca^2+^ chelator BAPTA by preincubating them in the presence of the permeable precursor BAPTA-AM (10 μM) for 10 min. It is noteworthy that VG Ca^2+^ currents in taste cells were usually prone to rapid rundown within 5–10 min. Fortunately, this interfering phenomenon could be slowed down sufficiently by incubating cells with the AC activator forskolin (0.1 μM), which elevated intracellular cAMP and that maintained the activity of VG Ca^2+^ channels [26]. As a result, most (~70%) of the forskolin-loaded type III cells exhibited sufficiently stable VG Ca^2+^ currents within 20 min at least, and these stabilized cells were further used to examine the dependence of VG Ca^2+^ entry on extracellular Ca^2+^ varied within 0.5–10 mM.

**Figure 2:**
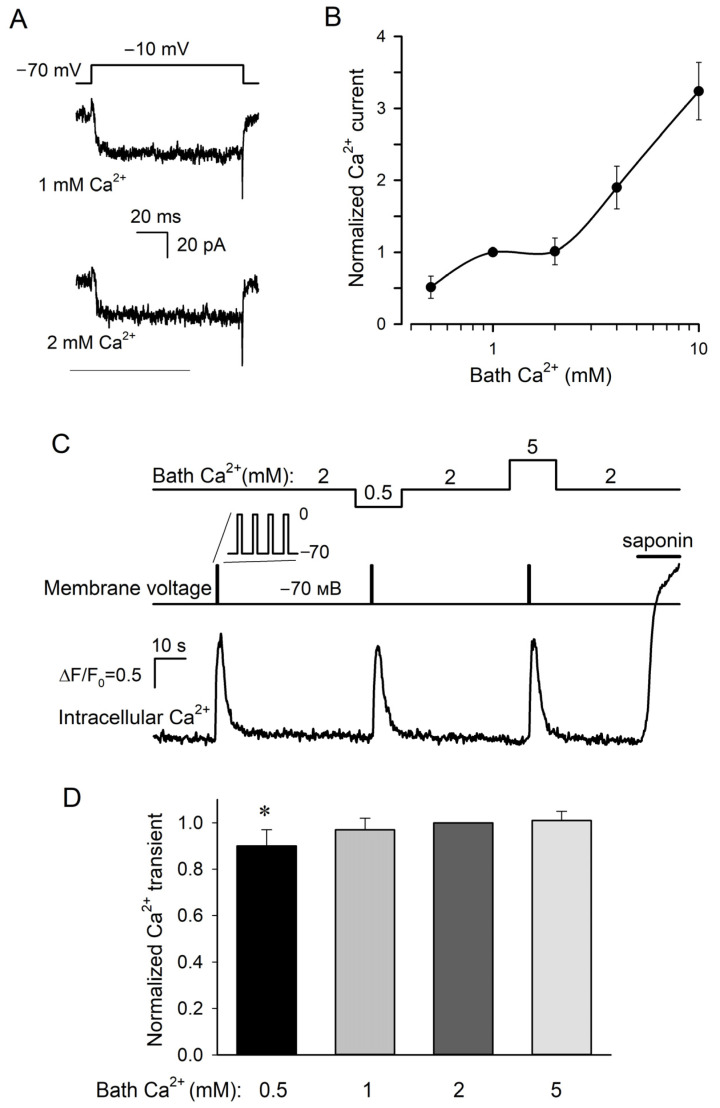
Dependencies of VG Ca^2+^ currents and Ca^2+^ transients on bath Ca^2+^. (**A**) Representative recording of Ca^2+^ currents (*n* = 49) at 1 and 2 mM Ca^2+^ in the bath using the P/4 protocol and perforated patch approach. The cell was held at −70 mV and polarized to −10 mV by 100 ms voltage pulses. The recording conditions as in Figure 1A, except that the cell was pretreated with 10 μM BAPTA-AM for 10 min and that 0.1 μM forskolin was present in the bath. (**B**) The dependence of sustained VG Ca^2+^ current at −10 mV (●) on bath Ca^2+^. This current was determined as a difference between integral currents recorded in control and in the presence of 50 μM Cd^2+^. The solid line is a spline approximant. (**C**) Representative monitoring (*n* = 17) of intracellular Ca^2+^ (bottom trace) in a patch-clamped type III cell loaded with Fluo-8 at varied bath Ca^2+^ (upper curve). The cell was held at −70 mV and depolarized to 0 mV for 20 ms every 50 ms during 1 s (middle curve). The recording conditions as in Figure 1A, except that 0.1 μM forskolin was present in the bath. Deviations of cytosolic Ca^2+^ from a resting level were quantified by the ratio ΔF/F_0_, where ΔF = F − F_0_, F is the instant Fluo-8 fluorescence, F_0_ is the averaged fluorescence at the very beginning of the recording. (**D**) Averaged Ca^2+^ transient elicited in type III cells by depolarization as in (**C**) at bath Ca^2+^ varied from 0.5 to 5 mM. In each particular experiment, Ca^2+^ transients at varied bath Ca^2+^ were normalized to a Ca^2+^ response at 2 mM Ca^2+^. The data are presented as the mean ± S.D. (*n* = 14–17). Unlike the Ca^2+^ burst at bath Ca^2+^ of 0.5 mM, the depolarization elicited Ca^2+^ transients at 1, 2, and 5 mM were statistically indistinguishable (*p* > 0.01, Student’s *t*-test). The asterisk means statistically representative difference (*p* = 0.001).

In total, we identified and assayed 71 cells, which exhibited well-resolved VG Ca^2+^ currents, and among them, 49 cells (69%) exhibited VG Ca^2+^ currents being unexpectedly subtly affected by the increase in bath Ca^2+^ from 1 to 2 mM (Figure 2A). Consequently, as a function of bath Ca^2+^, the current magnitude exhibited a plateau (Figure 2B). The previously elaborated models of ion transport involving ion channels suggest that in most cases, ion flux is determined largely by the binding of permeant ions to a single site within the channel pore [27]. This idea has been validated experimentally, particularly in the case of L-type Ca^2+^ channels, for which single-channel current *i* obeys the dependence [28]:

(1) $$i=i_{\max}\frac{a_{\mathrm{Ca}}}{a_{1/2}+a_{\mathrm{Ca}}}$$

where *a*_Ca_ is the activity of Ca^2+^ ions, *a*_1/2_ is the half-effect activity, and *i*_max_ is the single-channel current at *a*_Ca_ → ∞. For an integral current *I* mediated by *N* identical channels, it can be written as:

(2) I=iNP

where *P* is the open probability of the channel. Given that at low concentrations, ion activity is directly proportional to ion concentration, Equations (1) and (2) give the following dependence of the integral Ca^2+^ current on external Ca^2+^:

(3) $$I=I_{\max}P\frac{C}{C_{1/2}+C}$$

where *I*_max_ = *i*_max_*N*, *C* is the concentration of bath Ca^2+^, and *C*_1/2_ is the half-effect concentration. Equation (3) describes the monotonic saturating curve without any plateau, provided that the open probability is independent of Ca^2+^. Since here cytosolic Ca^2+^ was strongly buffered with BAPTA, which should have canceled the effects of intracellular Ca^2+^ on Ca^2+^ channel gating, the plateau in the experimental curve (Figure 2B) suggested that external Ca^2+^ somehow affected the open probability.

We also analyzed the correlation between extracellular Ca^2+^ and Ca^2+^ transients elicited by VG Ca^2+^ entry in type III cells. Being preloaded with Fluo-8, cells were patch clamped to identify type III cells with sufficiently active VG Ca^2+^ channels. Given that short depolarization elicited usually small and poorly resolved Ca^2+^ signals (Figure 1B), VG Ca^2+^ entry was stimulated by a 1 s train of AP-like voltage pulses that depolarized cells from −70 mV to 0 mV for 20 ms every 50 ms (Figure 2C, middle curve). In 17 out of 23 cells assayed in this manner (74%), depolarization induced more or less similar Ca^2+^ transients (Figure 2C,D) at different bath Ca^2+^ varied from 0.5 to 5 mM (Figure 2C, upper curve). Theoretically, the apparent constancy of depolarization-elicited Ca^2+^ signals, which were in fact dependent on bath Ca^2+^, might take place if VG Ca^2+^ entry saturated the Ca^2+^ dye in all cases. However, we excluded this possibility because the permeabilization of cells with saponin (10 μg/mL) always initiated much stronger Ca^2+^ signals compared to depolarizing voltage (Figure 2C, bottom trace).

To compare different recordings, a depolarization-evoked Ca^2+^ transient at a given bath Ca^2+^ was normalized to a Ca^2+^ response observed at 2 mM Ca^2+^ and then normalized Ca^2+^ signals associated with particular extracellular Ca^2+^ were averaged over 17 designated cells. It turned out that on average, the Ca^2+^ transients at 1 and 5 mM Ca^2+^ were statistically indistinguishable from the Ca^2+^ response at 2 mM Ca^2+^, which exceeded the Ca^2+^ transient at 0.5 mM Ca^2+^ solely by ~10% (Figure 2D). Thus, in most (74%) type III taste cells, Ca^2+^ entry stimulated by constant voltage originated Ca^2+^ transients, which remained virtually invariant at significantly varied Ca^2+^ in the bath. This phenomenon provided a rationale for further experimentation to clarify the dependence of neurotransmitter exocytosis driven by VG Ca^2+^ entry in type III cells on bath Ca^2+^.

### 3.2. Serotonin Release

#### 3.2.1. Serotonin Biosensor

**Figure 3:**
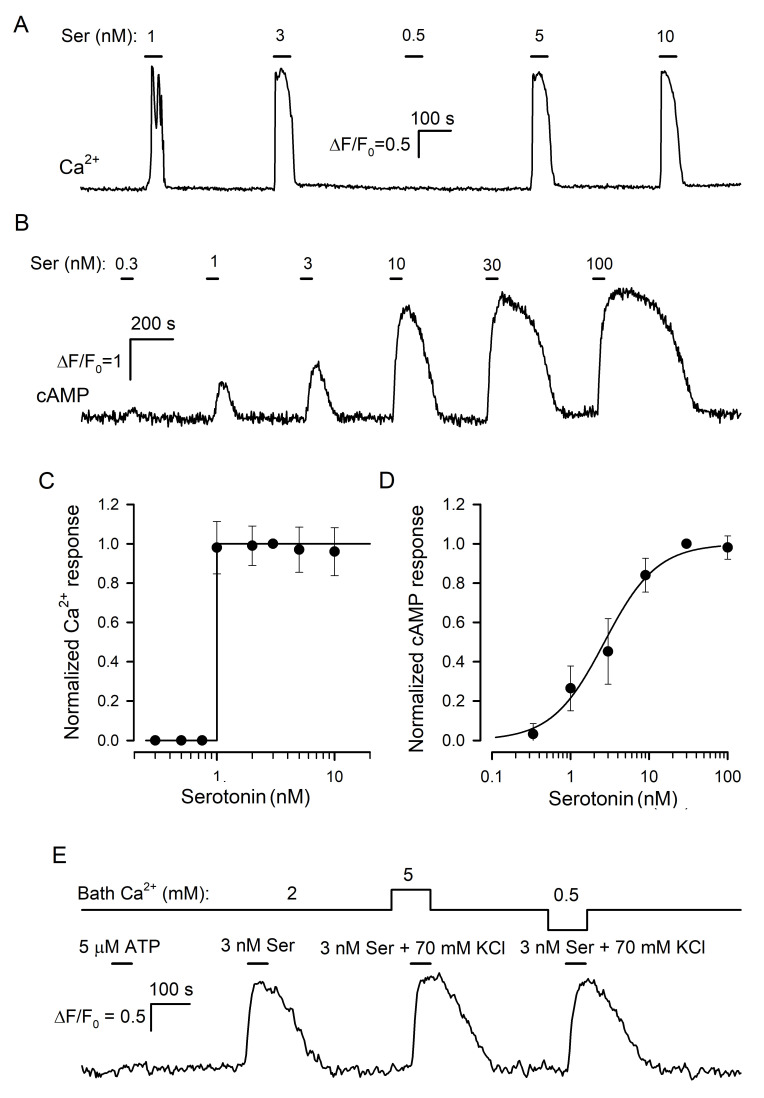
Serotonin biosensors. (**A**) Monitoring of intracellular Ca^2+^ by Fluo-8 fluorescence in a 5-HT_2C_-positive CHO cell sequentially stimulated by serotonin at the indicated doses. (**B**) Monitoring of intracellular cAMP with the Pink Flamindo sensor in a HEK-293 cell expressing the 5-HT_4_ receptor. Serotonin was applied serially as indicated. (**C**) Dose–response curve for the 5-HT_2C_-biosensor. In each particular experiment, a Ca^2+^ transient (ΔF/F_0_) elicited by serotonin at a given dose was normalized to a serotonin response at 3 nM. The data (cycles) are presented as the mean ± S.D. (*n* = 46). The solid line represents the Heaviside step function H(S-1) with S being serotonin concentration. (**D**) Dose–response curve for the 5-HT_4_/PF biosensor. In each particular experiment, cAMP responses to serotonin at varied doses were normalized to a cAMP transient elicited by 30 nM serotonin. The data (cycles) are presented as the mean ± S.D. (*n* = 31). The solid line represents the Hill equation R = S^n^/(S_0.5_^n^ + S^n^) for the normalized cAMP response R with the Hill coefficient *n* = 1.3 and the half-effect concentration S_0.5_ = 27 nM. (**E**) Representative control experiment (27 cells) indicating that the 5-HT_4_/PF biosensor was insensitive to micromolar ATP and that its responsiveness to serotonin was negligibly affected by bath Ca^2+^ and independent of a K^+^/Na^+^ ratio in the bath solution.

In previous studies of taste cells, the cellular biosensor methodology was effectively employed to assay the secretion of a variety of neurotransmitters identified in the taste bud, including serotonin [21], ATP [29,30], noradrenalin [31], GABA [32], and acetylcholine [33]. Central to this approach was a cell line that expressed appropriate surface receptors coupled to Ca^2+^ mobilization so that neurotransmitter release from a taste cell could be monitored online by a Ca^2+^ signal elicited in a nearby biosensor. Huang and co-workers [21] pioneered the demonstration of serotonin release from type III cells by using CHO cells stably expressing the recombinant 5-HT_2C_ receptor. They characterized this biosensor as responsive to nanomolar serotonin with Ca^2+^ transients and reported for them a gradual dose–response curve. Note that only a gradual serotonin biosensor would have allowed one to adequately assay the influence of extracellular Ca^2+^ on serotonin secretion. We, therefore, generated a CHO cell line with the stable expression of the recombinant mouse 5-HT_2C_ receptor (Section SI) and obtained several monoclones of 5-HT_2C_-positive cells, which generated Ca^2+^ signals in response to serotonin with the threshold of 1–3 nM (Figure 3A). It turned out, however, that individual 5-HT_2C_-positive CHO cells did not respond to serotonin gradually. Instead, they either were irresponsive to the agonist at low doses or generated very similar Ca^2+^ signals to the agonist at different concentrations above the threshold (Figure 3A,C).

Thus, in our hands, the CHO/5-HT_2C_ biosensor exhibited the “all-or-nothing” responsivity to serotonin in contrast to the gradual dose dependence of virtually the same biosensor described previously (Figure 1A in [21]). Presumably, Huang et al. [21] characterized populational rather than individual responsivity of 5-HT_2C_-positive CHO cells. Indeed, in our experiments, 5-HT_2C_-positive CHO cells exhibited distinct sensitivity to serotonin (i.e., different thresholds) and generated Ca^2+^ transients that were similar in a particular cell but varied by response lag and value, in terms of ΔF/F, from cell to cell (Figure S1A). By averaging such serotonin responses over a cell population, a gradual dose dependence could be easily obtained (Figure S1B).

As demonstrated earlier, “all-or-nothing” responsivity to agonists is also characteristic of mesenchymal stromal cells from human adipose tissue [34]. Several lines of evidence favored Ca^2+^-induced Ca^2+^ release (CICR) as a primary mechanism underlying the step-like dose dependence of agonist responses [35,36]. Since the CICR mechanism is apparently intrinsic to all cells expressing IP_3_- and ryanodine receptors [37,38], it might be expected that the “all-or-nothing” responsivity to serotonin would be characteristic of every biosensor cell expressing any 5-HT receptor coupled to the phosphoinositide cascade. Consistent with this idea, HEK-293 cells transfected with the 5-HT_2B_ receptor responded to serotonin with Ca^2+^ transients in an “all-or-nothing” manner (Figure S1C,D), as was also the case with 5-HT_2C_-positive cells (Figure 3A,C). We also tried 5-HT_1A_ and 5-HT_1B_ receptors but failed to obtain their coupling to Ca^2+^ mobilization in transfected HEK-293 cells (Figure S1E,F). It thus appeared that some other approach should have been employed to obtain a gradual serotonin sensor.

In the family of serotonin GPCRs, 5-HT_4_ is basically coupled to AC stimulation rather than to Ca^2+^ mobilization [39]. Given that cAMP signaling does not involve any trigger-like mechanism, such as CICR [40], we expected that in 5-HT_4_-positive cells, serotonin would elicit cAMP responses that increased gradually with agonist dose. We cloned 5-HT_4_ from the mouse brain and expressed it in HEK-293, which were preliminarily transfected with the genetically encoded cAMP sensor Pink Flamindo (PF) [22] (Section SI). We obtained and examined multiple monoclones of 5-HT_4_/PF-positive cells and found several of them to generate well-resolved cAMP transients in response to nanomolar serotonin applied shortly (Figure 3B). It turned out that 5-HT_4_/PF cells showed an appropriate gradual dependence of intracellular cAMP signals on serotonin concentration (Figure 3B,D). Importantly, cAMP responses altered subtly when bath Ca^2+^ was varied within 0.5–5 mM or if 140 mM NaCl + 5 mM KCl in the bath was replaced with 75 mM NaCl + 70 mM KCl (Figure 3E). By these features, the 5-HT_4_/PF sensor was quite appropriate for assaying serotonin release triggered noninvasively by KCl-induced depolarization at varied extracellular Ca^2+^, as described below.

#### 3.2.2. Effects of Bath Ca^2+^ on Serotonin Release

In the taste bud, solely type III cells produce and release serotonin [16]. Although basically, the biosensor approach allowed one to detect depolarization-induced serotonin release from individual type III cells (Figure S2), this level of the assay was not sufficiently effective because the enzymatic/low Ca^2+^ dissociation of taste buds rendered many taste cells poorly functional. In particular, solely a small (7–12%) fraction of isolated type III taste cells liberated serotonin reproducibly on three consecutive applications of 70 mM KCl, the minimal series necessary for the conclusive recordings (Figure S2). Therefore, we mainly examined serotonin release from individual taste buds, wherein taste cells remained more robust.

**Figure 4:**
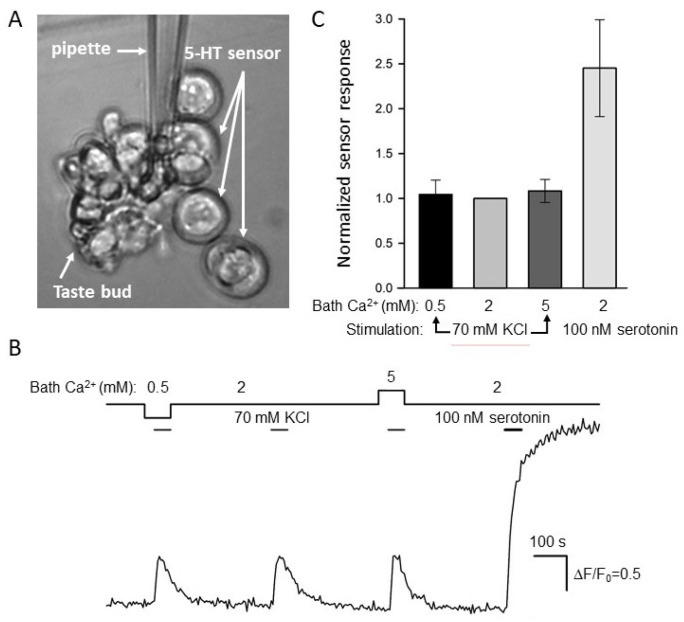
Biosensor assay of serotonin release. (**A**) Taste bud positioned nearby 5-HT_4_/PF-positive HEK-293 cells. (**B**) Evolution of the cAMP signal in the serotonin biosensor (the second 5-HT_4_/PF-cell from above) during the release assay in (**A**). The depolarization of taste bud cells by 70 mM KCl stimulated serotonin release that was apparently unaffected by a change in bath Ca^2+^ (solid line above the cAMP trace). The control application of exogeneous serotonin (100 nM) elicited a much higher response, indicating that the biosensor was not saturated by released serotonin. The deviations of cytosolic cAMP were quantified by the ratio ΔF/F_0_, where ΔF = F − F_0_, F is the instant fluorescence of the cAMP sensor Pink Flamindo, F_0_ is the averaged fluorescence at the very beginning of the recording. (**C**) Averaged cAMP responses of the 5-HT_4_/PF sensor upon taste bud depolarization as in (**B**) at bath Ca^2+^ of 0.5, 2, and 5 mM as well as by the stimulation with 100 nM serotonin. The data are presented as the mean ± S.D. (*n* = 22).

Initially, serotonin-sensitive 5-HT_4_/PF cells were put into a photometric chamber, wherein those were sedimented and attached to the Cell-Tak-coated bottom within 10 min. Next, several taste buds were sucked from a CV papilla into a glass pipette with an opening of ~70 μm and expelled into the camera. Being chosen as moderately dissociated, a taste bud was moved and fixed by a ~5 μm tip pipette in the immediate vicinity of a 5-HT_4_/PF sensor(s) (Figure 4A). The taste bud was shortly depolarized by 70 mM KCl at Ca^2+^ varied within 0.5–5 mM. Overall, 22 taste buds were assayed in this way, and all of them released serotonin on the depolarization, judging by responses of the serotonin sensor (Figure 4B), which per se was insensitive to the stimulation (Figure 3E).

It turned out that in each particular recording, serotonin sensor responses associated with the taste bud stimulation were more or less similar irrespective of bath Ca^2+^ (Figure 4B,C). Theoretically, the apparent independence of serotonin sensor responses of bath Ca^2+^ could occur if a stimulated taste bud released too much serotonin, thus saturating 5-HT_4_/PF cell responses in all cases. If so, biosensor signals would be virtually indistinguishable by value irrespective of bath Ca^2+^, even if the taste bud released different portions of serotonin in a bath Ca^2+^-dependent manner. To probe into this possibility, we finalized each particular recording with the application of 100 nM serotonin. This stimulation elicited a noticeable response of the 5-HT_4_/PF sensor, which invariably and markedly exceeded signals initiated by serotonin liberated from taste buds (Figure 4B,C). Thus, endogenously released serotonin never saturated the 5-HT_4_/PF sensor under our recording conditions, and therefore, the serially stimulated taste buds (Figure 4B) actually released more or less equal serotonin portions irrespective of bath Ca^2+^. Taken together, the abovementioned results (Figure 2 and Figure 4) argued for the existence of some regulatory circuit that rendered serotonin exocytosis to be only subtly dependent on extracellular Ca^2+^ within the surprisingly wide concentration range of 0.5–5 mM.

### 3.3. Evidence for the Involvement of CASR in the Regulation of Serotonin Release

We pondered on which machinery could stabilize neurotransmission in type III cells at varied Ca^2+^ in extracellular media and initially paid attention to the CICR mechanism for several reasons. First, CICR is known to contribute to neurotransmitter exocytosis [41,42,43,44]. Second, CICR has been implicated in shaping agonist-induced Ca^2+^ signals in a variety of cell types, making Ca^2+^ bursts universal kinetically and by value irrespective of the agonist dose above the threshold (Figure 3A) [35,36,45]. By analogy, we considered the possibility that in type III cells, CICR amplified and equalized initial Ca^2+^ signals originated by VG Ca^2+^ entry variable with bath Ca^2+^, thus making Ca^2+^ transients driving serotonin release to be virtually independent of external Ca^2+^ (Figure 4B,C). However, at constant bath Ca^2+^, depolarization-elicited Ca^2+^ transients varied with voltage (Figure 1B), following the I–V curve of the VG Ca^2+^ current (Figure 1A). This observation clearly indicated that at invariable bath Ca^2+^, CICR or any other intracellular mechanism was unable to counterbalance the obligatory dependence of an intracellular Ca^2+^ signal on the Ca^2+^ influx value. In contrast, the impulse depolarization of type III cells to invariable voltage, as should be the case with an AP train, produced similar Ca^2+^ transients at varied bath Ca^2+^ (Figure 2C,D). This suggested that the monitoring of extracellular Ca^2+^ was intrinsic to the regulatory machinery, which stabilized serotonin secretion at changeable Ca^2+^ (Figure 4B,C). Given that type III cells functionally express the extracellular Ca^2+^-sensing receptor (CASR) [46], this GPCR appeared to be an appropriate candidate for mediating a negative feedback regulation of serotonin release by external Ca^2+^. If so, CASR activation/inhibition should have impaired/facilitated serotonin secretion.

We, therefore, examined whether CASR ligands would be capable of affecting both depolarization-induced Ca^2+^ transients and associated serotonin release in type III cells. In particular, we probed some CASR ligands, including the natural agonist spermine and orthosteric synthetic agonist NPS R-568, as well as the CASR antagonist NPS-2143 [47]. Given that NPS R-568 and NPS-2143 are hydrophobic, we wondered whether these CASR ligands might modulate ion channels via membranotropic mechanisms not related to CASR activity. As a possible control indicative of nonspecific effects, we assayed an action of NPS R-568 and NPS-2143 on ion currents in type II cells, which did not express CASR [46]. It turned out that at the concentration of 0.5 μM close to EC_50_ ≈ 0.3–0.5 μM [48,49], NPS R-568 irreversibly diminished VG outward currents via non-selective CALHM channels in type II cells by 33 ± 5%, albeit the agonist negligibly affected VG Na^+^ currents (*n* = 8) (Figure S3A,B). These observations indicated that NSP R-568 could be capable of causing strong and irreversible effects on ion permeability via mechanisms not related to CASR stimulation. When applied at 2 μM, the dose being markedly above IC_50_ ≈ 0.04 μM [50], the CASR antagonist NPS-2143 subtly and reversibly decreased VG Na^+^ and outward currents in type II cells (*n* = 6) by 6–14% (Figure S3C,D). Although this result did not allow one to completely exclude a nonspecific action of NPS-2143 on the ion permeability of type III cells, this compound appeared to be a sufficiently specific CASR antagonist.

**Figure 5:**
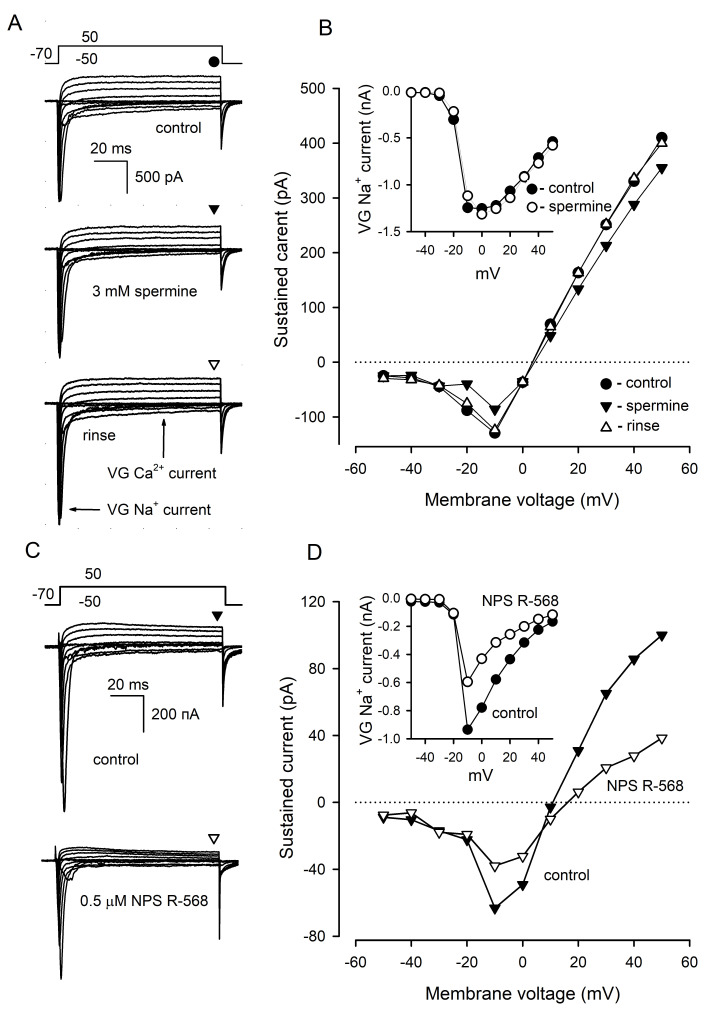
Effects of CASR agonists on VG currents in type III cells. (**A**) Representative (*n* = 9) recording of integral VG currents in a type III cell in control (upper panel) in the presence of 3 mM spermine (middle panel) and after rinse of the CASR agonist (bottom panel). The cell was held at −70 mV and polarized from −50 to 50 mV by 100 ms voltage pulses with 10 mV increment. Leak currents were subtracted using the P/4 protocol. The patch pipette contained 140 mM CsCl and amphotericin B (400 μg/mL), the bath solution contained 140 mM NaCl and 2 mM CaCl_2_. The sustained VG currents were evaluated in the moments indicated by the symbols above the current traces. (**B**). I–V curves of the sustained VG currents shown in (**A**). The inset, I–V curve for VG Na^+^ current recorded in control (●) and in the presence of 3 mM spermine (ο). (**C**) Representative effect of 0.5 μM NPS R-568 on VG currents in type III cells (*n* = 8). The recording conditions as in (**A**). (**D**) I–V curves of the sustained VG currents shown in (**C**). The inset, I–V curve for VG Na^+^ current recorded in control (●) and in the presence of 0.5 μM NPS R-568 (ο).

In type III cells (*n* = 9) dialyzed with 140 mM CsCl, 3 mM spermine reversibly inhibited VG Ca^2+^ currents by 41 ± 3% at −10 mV and outward, presumably anionic, currents by 13 ± 2% at 50 mV (Figure 5A,B), while VG Na^+^ currents were affected insignificantly (Figure 5B, inset). NPS R-568 (0.5 μM) diminished VG Ca^2+^ currents and outward currents by 44 ± 5% and 57 ± 7%, respectively (Figure 5C,D) (8 cells). In addition, VG Na^+^ currents were reduced by 34 ± 4% in the presence of NPS R-568 (Figure 5D, inset), which affected the mentioned ion currents irreversibly. Thus, compared to NPS R-568, which might exert rather non-specific effects on ion currents, spermine strongly diminished solely VG Ca^+^ currents in type III cells. We, therefore, inferred that the effect of spermine was specific; that is, mediated by CASR.

**Figure 6:**
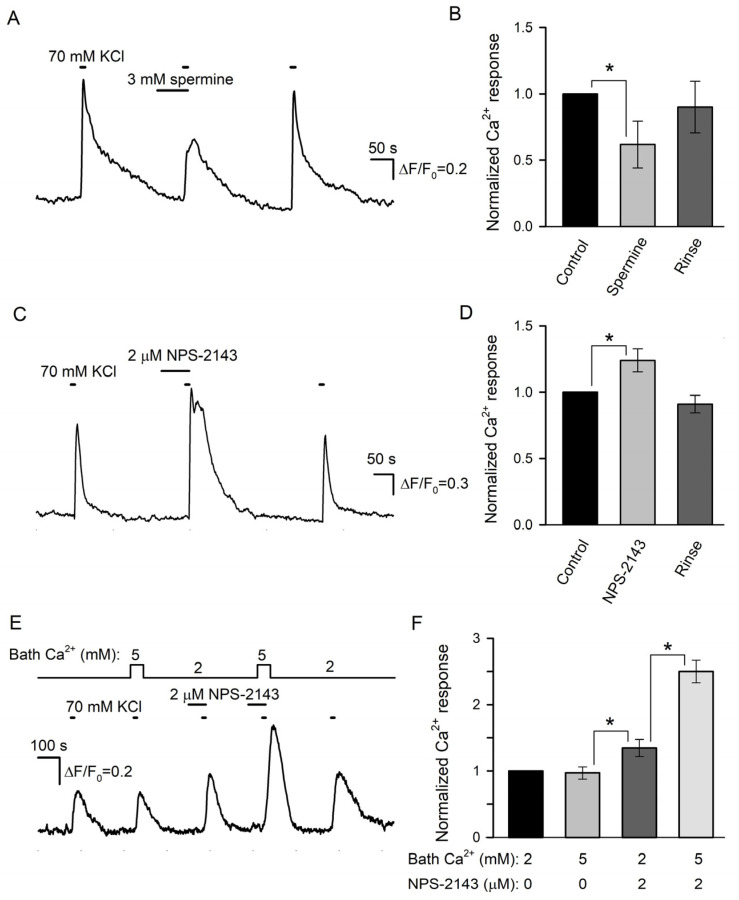
Effects of CASR ligands on depolarization-induced Ca^2+^ signals in type III taste cells. (**A**) Spermine (3 mM) reversibly diminished Ca^2+^ transients elicited by cell depolarization by 70 mM KCl. The cell was loaded with the Fluo-8 dye. (**B**) Averaged Ca^2+^ responses to 70 mM KCl recorded in control in the presence of spermine and after its rinse, as shown in (**A**). In each particular recording, the first control Ca^2+^ response was taken as a unit. The data are presented as the mean ± S.D. (*n* = 12). (**C**) 2 μM NPS-2143 enhanced Ca^2+^ response to 70 mM KCl. (**D**) Normalized and averaged Ca^2+^ responses (*n* = 9) in control in the presence of NPS-2143 and after its rinse. In all cases, the asterisk means statistically representative difference (*p* = 0.001). (**E**) NP-2143 (2 μM) made depolarization-induced intracellular Ca^2+^ transients dependent on extracellular Ca^2+^. (**F**) Normalized Ca^2+^ responses to 70 mM KCl at the bath Ca^2+^ of 2 or 5 mM in control and in the presence of 2 μM NPS-2143. The data are presented as the mean ± S.D. (*n* = 6). In all cases, the asterisk means statistically representative difference (*p* = 0.001).

We next examined the effects of spermine and NPS-2143 on Ca^2+^ responses initiated by the depolarization of individual taste cells by 70 mM KCl. Unfortunately, the responsivity of isolated type III cells to the depolarization was liable to rundown that was strengthened by the CASR ligands to some extent. For this reason, the effects of spermine and NPS-2143 on the same cell were poorly conclusive, and cell sensitivity to each compound was assayed separately. Overall, we treated 12 sufficiently robust type III cells with 3 mM spermine and found this CASR agonist to reversibly diminish depolarization-induced Ca^2+^ bursts by 38 ± 17% compared to control (Figure 6A,B). In contrast, KCl-induced Ca^2+^ transients were increased by 24 ± 8% in the presence of 2 μM NPS-2143 (9 cells) (Figure 6C,D).

In a designated experimental series, we tried to elucidate whether NPS-2143 could disrupt the equalization of Ca^2+^ transients elicited by the same depolarization of type III cells at different bath Ca^2+^ (Figure 2C). The appropriate assay required robust cells capable of generating multiple, at least four, reproducible Ca^2+^ responses to the stimulation. Although the responsivity of many cells was liable to rundown, finally, 6 cells were found to be suitably stable for conclusive recordings. Consistently with electrophysiological experiments (Figure 2C), the first two control stimulations by KCl elicited apparently similar Ca^2+^ transients irrespective of bath Ca^2+^ of 2 or 5 mM (Figure 6E,F). NPS-2143 (2 μM) not only expectedly sensitized cells (Figure 6C) but also made intracellular Ca^2+^ transients mediated by VG Ca^2+^ entry to be markedly dependent on extracellular Ca^2+^ (Figure 6E,F): in the presence of the CASR antagonist, Ca^2+^ responses at 2 and 5 mM Ca^2+^ were distinct by a factor of 1.85 ± 0.16 on average (Figure 6F). Taken together, the abovementioned observations (Figure 6) supported the idea that CASR could be involved in the negative regulation of VG Ca^2+^ entry, and by that, in the control of neurotransmitter exocytosis.

**Figure 7:**
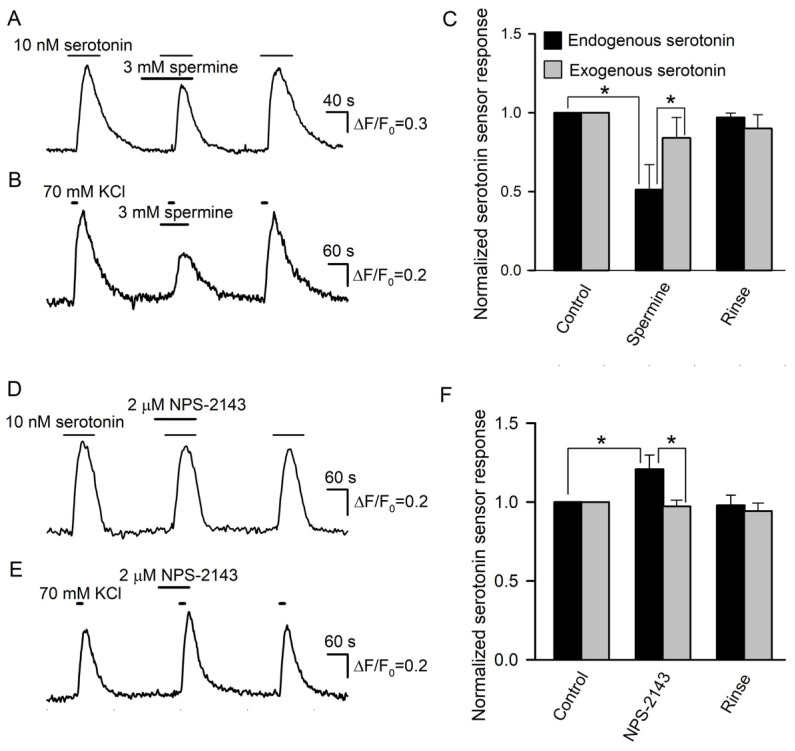
Effects of CASR ligands on biosensor responsivity and serotonin release. (**A**) Serotonin sensor responses in control, in the presence of 3 mM spermine, and after its rinse. (**B**) Effect of 3 mM spermine on depolarization-induced serotonin release assayed as in Figure 4A. (**C**) Averaged serotonin sensor responses to serotonin, either endogenously released from taste buds (*n* = 15) (black bars) or externally applied at 10 nM (*n* = 56) (gray bars), in control, in the presence of 3 mM spermine, and after its rinse. In each particular recording, the first control response was taken as a unit. The data are presented as the mean ± S.D. (**D**) NPS-2143 (2 μM) subtly affected serotonin sensor responsivity to exogenous serotonin applied at 10 nM. (**E**) Biosensor response to serotonin released from a taste bud was enhanced in the presence of 2 μM NPS-2143. (**F**) Averaged serotonin sensor responses to serotonin, either endogenously released from taste buds (*n* = 14) (black bars) or externally applied at 10 nM (*n* = 48) (gray bars), in control, in the presence of 3 mM spermine, and after its rinse. The data are presented as the mean ± S.D. In all cases, the asterisk means statistically representative difference (*p* = 0.001).

Prior to the assay of the expected modulation of serotonin secretion by spermine and NPS-2143, we performed control experiments to make clear whether these CASR ligands could influence the serotonin responsivity of the 5-HT_4_/PF sensor. It turned out that compared to the control, 3 mM spermine decreased cAMP signals in 5-HT_4_/PF cells elicited by 10 nM serotonin by 16 ± 13% on average (*n* = 56) (Figure 7A). NSP-2143 (2 μM) reduced biosensor responses solely by 4 ± 3% (*n* = 48), but this small effect was poorly reversible (Figure 7D). Thus, the 5-HT_4_/PF biosensor turned out to be somewhat sensitive to the CASR ligands.

Next, we examined how effectively the CASR agonist and antagonist could affect serotonin secretion in taste buds. Overall, 29 taste buds were assayed, and in control, 70 mM KCl stimulated robust serotonin release in all of them. When certain taste buds (*n* = 15) were pretreated with 3 mM spermine, their stimulation with KCl triggered biosensor responses that were reduced by 48 ± 16% on average compared to the control (Figure 7B,C). Although this effect was partly associated with the ~16% drop in sensor responsivity induced by 3 mM spermine (Figure 7A), the difference between averaged biosensor responses to endogenous and exogenous serotonin observed in the presence of the CASR agonist was statistically significant (Figure 7C). This suggested that the CASR agonist truly suppressed serotonin secretion by nearly 30%. In 14 taste buds treated with 2 μM NPS-2143, depolarization-induced serotonin release was increased by 21 ± 9% compared to the control (Figure 7E,F). This effect was sufficiently high and contrasted to the subtle inhibitory action of NPS-2143 on the serotonin responsivity of the 5-HT_4_/PF sensor (Figure 7D). Thus, the inhibition of CASR truly enhanced serotonin secretion. The described effects of the CASR ligands (Figure 7) provided a rationale to believe that CASR was indeed involved in a negative feedback regulation of serotonin release in type III taste cells.

## 4. Discussion

Unlike in vitro conditions with virtually unlimited extracellular volume, the important factor of cell physiology in biological tissues is the rather restricted intercellular space. In resting cells, ion fluxes through ion channels, including Na^+^ and Ca^2+^ entry and K^+^ release, are properly balanced by ion pumps, so that extracellular ions are maintained at constant levels. The impact of diverse stimuli on cells commonly entails a change in the activity of ion channels that can alter external ion composition essentially because the intercellular medium is usually smaller than the cell cytosol. This problem was recognized a long time ago in the case of brain tissues, wherein neuronal activity could transiently elevate extracellular K^+^ by several times, thereby depolarizing the resting potential of neurons and changing their excitability [51,52]. In search for mechanisms that could mediate the clearance of K^+^ ions accumulated in the extracellular medium, glial cells have been implicated in the removal of excess K^+^ [53,54]. Several concerted transport mechanisms presumably mediate K^+^ clearance, involving specialized isoforms of inward rectifying K^+^ channels, Na^+^/K^+^ ATPase, and Na^+^/K^+^/Cl^−^ cotransporter [54].

Here we focused on a somewhat similar problem associated with the homeostasis of extracellular Ca^2+^ in biological tissues. Evidence exists that Ca^2+^ in the intercellular medium can markedly fluctuate due to the electrical activity of cells, thereby affecting their physiology. It is particularly noteworthy that brief trains of synaptic transmission can induce transient depletion of extracellular Ca^2+^ in hippocampal area CA1 caused by Ca^2+^ entry through activated NMDA receptors. This activity-dependent drop in extracellular Ca^2+^ has been suggested to reflect a certain form of retrograde modulation of synaptic signaling in the brain [55].

The taste bud represents a tightly packed cell population [17]. Our estimates (see Introduction) suggested that if a given taste cell absorbs external Ca^2+^ to elevate cytosolic Ca^2+^ solely by ~100 nM, this could deplete Ca^2+^ in the nearby extracellular medium by ~1 mM. In a neighboring cell, this nearly two-fold drop in external Ca^2+^ would proportionally reduce Ca^2+^ influx. If this suggested scenario is valid, the secretion of neurotransmitters triggered by VG Ca^2+^ entry in a type III cell should not only depend on taste stimulation but also be influenced by the electrical activity of neighboring taste cells. The last would add a false informational flux to the neurotransmission between type III cells and afferent endings. We, therefore, surmised the existence of some machinery capable of stabilizing synaptic transmission in type III cells at fluctuating Ca^2+^ in the intercellular space. We verified this suggestion by analyzing how physiologically appropriate changes in bath Ca^2+^ could affect three associated parameters, including VG Ca^2+^ currents, Ca^2+^ transients elicited by VG Ca^2+^ entry, and depolarization-induced secretion of serotonin in isolated type III cells. It turned out that these parameters showed weak or negligible sensitivity to bath Ca^2+^ varied in a certain range of concentrations (Figure 2 and Figure 4). These findings strongly supported the idea that type III cells employ a special regulatory machinery to stabilize the neurotransmission at unstable extracellular Ca^2+^.

It should be noted that here we predominantly assayed taste cells isolated from the CV papilla, although in the limited number of experiments, foliate taste cells were examined as well. Expectedly, type III foliate cells also exhibited the invariancy of serotonin release at varied bath Ca^2+^ (Figure S4). Given that, compared to CV and foliate taste cells, the assay of individual cells from the fungiform taste papilla is much more technically complicated (e.g., [41]), this cell population was not explored in the present study. The question, therefore, remains whether the phenomenon, which was validated for CV and foliate type III cells (Figure 4 and Figure S4), also exists in fungiform taste cells.

We pondered on the mechanism(s) that could provide the appropriate regulation of the neurotransmission in type III cells. Among possible scenarios, we considered the CICR mechanism, which has been implicated in the regulation of neurotransmitter exocytosis in a variety of different cells [42,43,44]. It was suggested that by amplifying and equalizing initial Ca^2+^ transients elicited by VG Ca^2+^ entry in type III cells, CICR could provide the apparent independence of serotonin release on extracellular Ca^2+^ (Figure 4B,C). However, at constant bath Ca^2+^, depolarization-induced Ca^2+^ transients varied with voltage (Figure 1C), and therefore were dependent on a Ca^2+^ influx value (Figure 1B,D). This observation indicated clearly that CICR alone was unable to level off intracellular Ca^2+^ transients produced by VG Ca^2+^ entry at different voltages. In contrast, at constant depolarization but variable Ca^2+^ in the bath, resultant Ca^2+^ signals in the cell cytosol and serotonin release were virtually immune to Ca^2+^ fluctuations (Figure 2C,D). This finding pointed out that a sought regulatory mechanism should have detected extracellular Ca^2+^ to properly adjust Ca^2+^ entry and, perhaps, the exocytosis machinery as a whole. Given that CASR is functionally expressed in type III cells [46], this GPCR might serve as a sought detector of extracellular Ca^2+^ as well as a regulator of serotonin exocytosis in these cells. In support of this idea, the CASR activation with spermine decreased both Ca^2+^ transients originated by VG Ca^2+^ entry and serotonin secretion, while the CASR inhibition with NPS-2143 facilitated these processes (Figure 6 and Figure 7).

Reportedly, CASR is involved in the regulation of neuronal excitability, neurotransmission, and synaptic plasticity [56,57]. It was particularly shown that the CASR agonist spermidine reduced the efficacy of synaptic transmission in cortical neurons, which, in contrast, was markedly enhanced by the genetic ablation of CASR [58]. Being a pleiotropic GPCR, CASR can be coupled by heterotrimeric G_q/11_, G_i/o_, G_12/13_, and G_s_ proteins and β-arrestins to a variety of effectors and signaling pathways. Ubiquitously engaged are adenylyl cyclase producing cAMP and phospholipase C (PLC) hydrolyzing phosphatidylinositol 4,5-bisphosphate (PIP_2_) to generate two second messengers, inositol 1,4,5-trisphosphate (IP_3_) and diacylglycerol (DAG) [59,60].

Among intracellular molecules involved in CASR signaling, many are documented as regulators of VG Ca^2+^ channels. The gating of these channels is particularly modulated by direct interaction with G proteins, Ca^2+^/calmodulin, lipids, and fatty acids as well as by phosphorylation mediated by a number of protein kinases, including PKA, PKC, and CaMK regulated by cAMP, Ca^2+^/DAG, and Ca^2+^/calmodulin, respectively [61,62,63,64]. CASR can regulate neurotransmitter exocytosis not only by affecting Ca^2+^ influx via VG Ca^2+^ channels but also by controlling the level of PIP_2_ that serves as a hub for proteins, which govern the turnover of presynaptic vesicles [65,66,67]. In addition, by engaging PLC, CASR could control the production of DAG that regulates exocytosis by binding to Munc13-1 and via PKC-mediated phosphorylation of Munc18-1, the proteins orchestrating the formation of the SNARE complex that ensures vesicle fusion [68,69,70]. Thus, theoretically, multiple pathways could couple CASR to serotonin exocytosis in type III cells.

The analysis of the CASR coupling to VG Ca^2+^ channels is a complicated issue because VG Ca^2+^ currents in type III cells are usually small under physiological conditions (Figure 1A). Although with 10 mM Ca^2+^ in the bath, VG Ca^2+^ currents were enhanced by nearly three times compared to the normal conditions (Figure 2B), type III cells poorly tolerated a prolonged treatment with so high extracellular Ca^2+^. In addition, characteristic of CASR-mediated responsivity of cells to extracellular Ca^2+^ are high cooperativity and EC_50_ ≈ 2.5 mM [46,71]. Consequently, 10 mM Ca^2+^ should have elicited maximal CASR activity in type III cells, thus saturating a regulatory circuit downstream of CASR and making its sensitivity to inhibitors of effectors involved presumably abnormal. In any way, a particular compound could be considered as a probe for the CASR-mediated circuit only if its effect on cell functions, VG Ca^2+^ currents in the given case, would be influenced by CASR ligands. We, therefore, inferred that the detailing of the signaling circuit, which couples CASR to VG Ca^2+^ entry and, more generally, to serotonin release, would require special efforts, including multiple recordings from sufficiently robust taste cells occurring rarely. As highly complicated and laborious, the appropriate experiments were not carried out in this work but suggested for the future.

To conclude, our overall findings strongly argue for the existence of a CASR- mediated circuit that stabilizes serotonin secretion in type III cells at fluctuating Ca^2+^ in the extracellular medium. VG Ca^2+^ channels appear to be under the control of a negative feedback that regulates the neurotransmitter exocytosis in an extracellular Ca^2+^-dependent manner. Given, however, that VG Ca^2+^ currents were invariant in the relatively narrow range of bath Ca^2+^ concentrations of 1–2 mM (Figure 2B), the CASR-mediated mechanism should involve some other intracellular pathways to level off serotonin secretion within 0.5–5 mM Ca^2+^ (Figure 4). The involved regulatory circuit remains to be elucidated.

## Data Availability

Not applicable.

## Supplementary Materials

The following supporting information can be downloaded at: https://www.mdpi.com/article/10.3390/cells11081369/s1, Figure S1: Responsiveness of cells transfected with different 5-HT receptors; Figure S2: Release of serotonin from an individual CV cell of the type III assayed with the biosensor approach; Figure S3: Effects of CASR ligands on VG currents in type II cells; Figure S4: Assay of serotonin release from a foliate taste bud with the 5-HT4/PF-biosensor at varied bath Ca^2+^.
