# Supplementary material for: Taste Cells of the Type III Employ CASR to Maintain Steady Serotonin Exocytosis at Variable Ca2+ in the Extracellular Medium
Source: Cells. 2022 Apr 18;11(8):1369. doi: 10.3390/cells11081369 (PMC9030112; doi:10.3390/cells11081369)
Supplement: Supplementary file 1 [file cells-11-01369-s001.zip › cells-1653004-supplementary.pdf]

cells

MDPI

SUPPLEMENTARY MATERIALS

I. Serotonin biosensors.

The cellular biosensor approach is effective for the on-line monitoring of secretion of signaling molecules from different cells, including taste cells. Central to this approach is a cell line that expresses appropriate surface receptors that are coupled to any intracellular signal, usually to Ca^{2+}mobilization, which could be easily visualized. To adapt this approach for exploring serotonin release from taste cells, we cloned and heterologously expressed a variety of 5-HT receptors, including the mouse 5-HT_{2C}, 5-HT_{1A}, 5-HT_{1B}, and 5-HT_4 receptors as well as the human 5-HT_{2B} receptor.

I.1. Molecular cloning of 5-HT receptors.

For cloning 5-HT receptors, RNA was isolated from the mouse brain or from mesenchymal stem cells (MSCs) derived from the human adipose tissue by using RNeasy mini kit (Qiagen). Next, isolated RNA was reverse-transcribed with SuperScript IV reverse transcriptase (Invitrogen) and random hexamer primers. For PCR amplification of coding sequences of 5-HT receptors, PhusionHot Start II DNA Polymerase (ThermoFisher Scientific) was used.

5-HT_{2C}. The coding sequence of 5-hydroxytryptamine receptor 2C (Htr2c, NM_008312.4) encompassing nucleotides 689-2065 was cloned from mouse brain by RT-PCR with the primers TAAGAATTCTGGCCACCATGGTGAACCTGGCACT and TATGTCGACTGCACACTACTAATCCTCTCGCTG and subcloned into EcoRI and SalI sites of the pDsRed-Monomer-N1 mammalian expression vector in-frame with DsRed-Monomersequence.

5-HT_{2B}. The coding sequence of 5-hydroxytryptamine receptor 2B (HTR2B, NM_000867.5) wasamplified by RT-PCR from human MSCs with the primers GAGAAGCTTCAGCAAATGGCTCTCTCTTACAGA and GAGGTCGACTGTACATAACTAACTTGCTCTTCA. The fragment encompassing nucleotides432-1880 was cloned into HindIII and SalI sites of the pDsRed-Monomer-N1 vector.

5-HT_{1A}. The coding sequences of Mus musculus 5-hydroxytryptamine receptors 1A (Htr1a) werecloned by RT-PCR from mouse brain and inserted into the HindIII site of the pDsRed-Monomer-N1 vector by seamless cloning with In-Fusion® HD Cloning Kit according to the manufacturer (Takara). Primers for amplification of Htr1a sequence corresponding to nucleotides 556-1824 of NM_008308.4 were TCTCGAGCTCAAGCTTGCAGGCATGGATATGTTCAGTCT and GCAGAATTCGAAGCTTGCGGCAGAACTTGCACTTGA.

5-HT_{1B}. The coding sequences of Mus musculus 5-hydroxytryptamine receptors 1B (Htr1b) werecloned by RT-PCR from mouse brain and inserted into the HindIII site of the pDsRed-Monomer-N1 vector by seamless cloning with In-Fusion® HD Cloning Kit according to the manufacturer (Takara). Primers for amplification of Htr1b sequence corresponding to nucleotides 461-1624 of NM_010482.2 were TCTCGAGCTCAAGCTTAGAGCTATGGAGGAGCAGGGT and GCAGAATTCGAAGCTTACCTGCGCACTTAAAGCGT.

5-HT_4. Mus musculus 5-hydroxytryptamine receptor 4 (Htr4) was cloned by RT-PCR from mouse brain with primers TATAAGCTTCCTGTAATGGACAAACTTGATG and TATAAGCTTAGTATCACTGGGCTGAGC. The fragment encompassing nucleotides 207-1376of NM_008313.4 was subcloned into the HindIII site of the pAcGFP1-Hyg-N1 vector in-frame with GFP sequence.

The constructs pDsRed-Monomer-N1/5-HT2C, pDsRed-Monomer-N1/5-HT2B, pDsRed- Monomer-N1/5-HT1A, pDsRed-Monomer-N1/5-HT1B, and pAcGFP1-Hyg-N1/5-HT4 were confirmed by restriction enzyme digestion and sequencing.

I.1. Heterologous expression of 5-HT receptors.

The recombinant receptor 5-HT2C was expressed in CHO cells, while 5-HT1A, 5-HT1B, 5-HT2B , and 5-HT4 were expressed in HEK-293 cells. The cells of the HEK-293 and CHO lines were routinely cultured in the appropriate growth medium (1 ml) containing Dulbecco's modified Eagle's (DMEM) medium or the F12 medium (both from Invitrogen), respectively, 10%(v/v) fetalbovine serum (HyClone), glutamine (1 %), and the antibiotic gentamicin (100 µg/ml) (Invitrogen)in 12-well culture plates. Cells were grown in a humidified atmosphere (5 % CO_2 and 95 % O_2) at37 °C. Before the day of transfection, cells were put in 12-well culture plates at the density of 3– 5 $\times 10^5$ cells. The transient transfection was performed by adding 100 µl of OPTI-MEM (Gibco) containing plasmid DNA (1 µg) and FuGENE 6 (4 µl) (Promega) to cultured cells. After 12-h incubation, the transfection mixture was replaced with the growth culture medium. Basically, cellswere assayed physiologically 24–72 h after transfection.

I.1.1. Monoclones of 5-HT2C-positive cells.

To generate monoclones of 5-HT2C-positive cells, CHO cells transfected with the pDsRed- Monomer/5-HT2C construct were maintained in the F12 growth medium supplemented with 0.6 mg/ml G-418 for the following 3 weeks. The antibiotic-resistant cells were separated by their DsRed fluorescence at 590 nm using a FACS Aria SORP sorter (Beckton Dickinson), and those exhibiting most intensive red fluorescence were collected individually for further culturing in a 96-vell plate. Overall, 53 monoclones were obtained from individual 5-HT2C-positive cells, and those were further maintained in the presence of 0.3 mg/ml G-418. Each monoclone was screenedfor functionality with Ca^{2+} imaging, and 5 monoclones of CHO/5-HT2C cells were selected for further experimentation based on their responsiveness to nanomolar serotonin.

I.1.1. Monoclones of 5-HT4/PF cells.

Prior to the generation of a cell line stably expressing the 5-HT4 receptor, we produced cells stably expressing the genetically encoded cAMP sensor Pink Flamindo (Harada et al., 2017). HEK-293 cells were transfected with the Pink Flamindo plasmid (a gift from Tetsuya Kitaguchi,Addgene plasmid # 102356; http://n2t.net/addgene:102356; RRID:Addgene_102356) by replacing the growth medium with the transfection mixture, containing plasmid DNA (1.6 µg) and Lipofectamine 3000 (4 µl) per 1 ml of serum-free DMEM. After 24-h incubation, the transfection mixture was replaced with the normal culture medium, and transfected cells were cultured in the presence of 0.6 mg/ml G-418 (Invivogen) for 3 weeks. The antibiotic-resistant cells were analyzed using the FACS Aria SORP sorter (Beckton Dickinson), and those exhibitingmost intensive Pink Flamindo fluorescence at 590 nm were collected individually for further culturing in a 96-well plate. Overall, individual Pink Flamindo (PF)-positive cells originated 63cellular monoclones, which were further maintained in the presence of 0.3 mg/ml G-418. Each monoclone was screened for functionality with cAMP-imaging, and 7 monoclones of HEK- 293/PF cells were selected as generating sufficiently high cAMP responses to 10 µM forskolin.

Next, HEK-293/PF cells were transfected with the plasmid vector pAcGFP1-Hyg-N1/5-HT4as described above, and then they were subjected to the antibiotic selection in the presence of G418(0.3 mg/ml) and Hygromycin B Gold (0.1 mg/ml) (Invivogen). Selected by Pink Flamindo and GFP fluorescence with the FACS Aria SORP sorter, 5-HT4/PF-positive cells were cultured individually to originate monoclones. Of 72 5-HT4/PF monoclones obtained, each was functionally assayed, and 4 monoclones were selected as generating well-resolved intracellular cAMP signals in response to nanomolar serotonin (Fig.3B in the main text).

I.1. Functional assay of 5-HT receptors coupled to Ca^{2+} mobilization

The serotonin responsivity of cells expressing recombinant 5-HT_{2C}, 5-HT_{2B}, 5-HT_{1A}, or 5-HT_{1B} receptors was characterized with Ca^{2+} imaging. Assayed cells were plated onto a photometric chamber of nearly 150 µl volume. The last was a disposable coverslip with attached ellipsoidal resin wall. The chamber bottom was coated with Cell-Tak (Corning) ensuring strong cell adhesion.Attached cells were loaded with the Ca^{2+} dye at room temperature (23–25 °C) by incubating themwith Fluo-8AM (4 µM) and Pluronic (0.02%) for 20 min followed by the 30-min rinse with the bath solution (mM): 140 NaCl, 5 KCl, 1 $MgCl_2$, 2 $CaCl_2$, 10 HEPES-NaOH (pH 7.4), 10 glucose.

Individual CHO cells stably expressing 5-HT_{2C} responded to serotonin in an "all-or-nothing" manner: they either were irresponsive to the agonist at low doses or generated very similar Ca^{2+} signals to the agonist at different concentrations above the threshold (Fig.S1A, upperpanel). Note that sensitivity to serotonin, response lag and response magnitude, in terms of $\Delta F/F_0$,varied from cell to cell. As a result, when serotonin responses of a population of CHO/5-HT_{2C} cells were averaged by summarizing fluorescent signals of individual cells and representing the total fluorescence of a cell population as $\Delta F/F_0$ (Fig.S1A, bottom panel), the resultant dose- response curve became gradual (Fig.S1B).

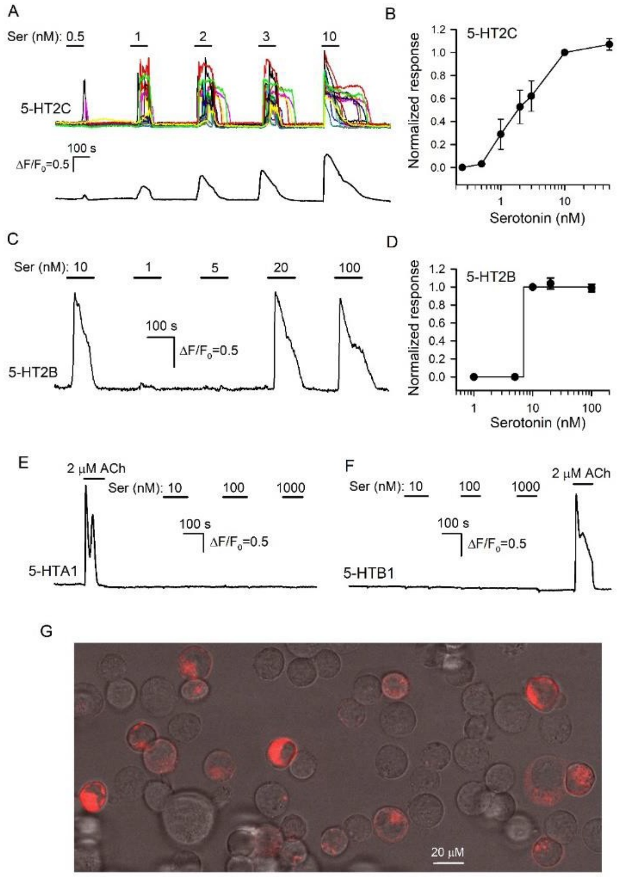

Figure S1. Responsiveness of cells transfected with different 5-HT receptors. (A) Upper panel, the concurrent monitoringof intracellular Ca^{2+} in 5-HT_{2C}-positive CHO cells loaded with Fluo-8; 20 out of 86concurrently assayed cells were arbitrarily chosen. The cells were stimulated byserotonin at different doses as indicated.The deviation of cytosolic Ca^{2+} from a resting level in a particular cell was quantified by the ratio $\Delta F/F_0$, where $\Delta F = F\text{-}F_0$, F is the instant Fluo-8 fluorescence, F_0 is the averaged fluorescence in the very beginning of therecording. The bottom panel, the populational Ca^{2+} trace that was generated by averaging 86 individual fluorescence traces being similar to those shown in the upper panel. (B) Dose-responsecurve generated by averaging populational serotonin responses of 5-HT_{2C}-cells over 5 experiments, including one shown in (A). The cellresponse was calculated as $R=\Delta F/F_0$, where $\Delta F = F_{max}\text{-}F_0$, F_0 is cell fluorescence measuredimmediately before seroto-

nin application, F_{max} is the fluorescence peak characteristic of a given serotonin response. The averaged response to 10 nM serotonin was taken as a unit. The data are presented as a mean±S.D. (n=5). (C) Serotonin responses of 5-HT_{2B}-positive HEK-293 cellsstimulated serially as indicated. (D) Dose responsecurve for the 5-HT_{2B}-biosensor. For each particular 5-HT_{2B}-cell, a Ca^{2+} transient ($\Delta F/F_0$) elicited by serotonin at a given dose was normalized to a response to 3 nM serotonin. The data (cycles) are presented as a mean±S.D. (n=38).The solid line represents the Heaviside step function H(S-7) with S being serotonin concentration. (E, F) Serotonin never stimulated Ca^{2+} signaling in HEK-293 cells, which heterologously expressed the 5-HT_{1A} receptor (E) or 5-HT_{1B} receptor (F). (G) Images of HEK-293 cells transiently transfected with the 5-HT_{1A} receptor fused with the red fluorescent protein DsRed.

Being transiently transfected with the $5HT_{2B}$ receptor, HEK-293 cells also responded to serotonin with Ca^{2+} transients in the "all-or-nothing" manner (Fig.S1C, D). In contrast, we failed to observe serotonin-induced Ca^{2+} signaling in HEK-293 cells transiently transfected with $5HT_{1A}$(Fig.S1E) or $5HT_{1B}$ (Fig.S1F). It is noteworthy that fluorescence of the DsRed marker fused withthe receptor protein was apparently located in the plasmalemma in many cells (Fig.S1G). Thus, although recombinant $5HT_{1A}$ and $5HT_{1B}$ receptors were apparently transported on the cell surface,they did not endow transfected cells with responsivity to serotonin, in terms of Ca^{2+} signaling, presumably because these recombinant serotonin GPCRs were not coupled to the phosphoinositidecascade in HEK-293 cells.

I. Assay of individual taste cells with the serotonin biosensor.

Basically, the biosensor approach allowed for detecting depolarization-induced serotonin release from individual cells of the type III from the CV papilla, if they were sufficiently robust. In the representative successful experiment (n=7) illustrated in Fig.S3, a taste cell, which was identifiedas type III by serotonin release, was stimulated by 70 mM KCl in series. Synchronously with depolarizing stimulation, the serotonin sensor generated pretty similar responses visualizingtransient serotonin release. The control application of exogeneous serotonin (10 nM) elicited the markedly higher response, showing that the biosensor was not saturated by released serotonin. This observation indicated that in robust cells of the type III, the KCl-induced serotonin release was reproducible in a series of 3 stimulation at least.

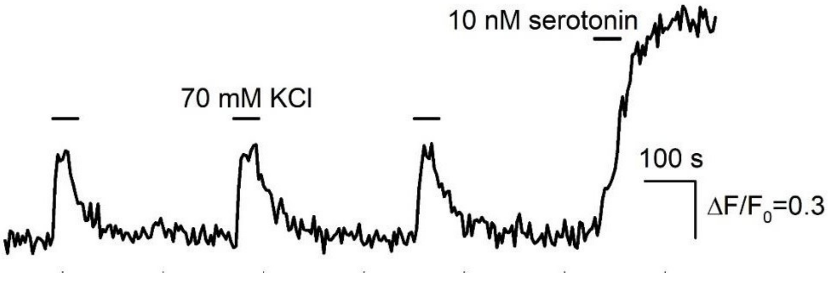

Figure S2. Release of serotonin from an individual CV cell of the type III assayed with the biosensor approach. The noisy experimental trace represents the cAMP signal in a nearby 5- HT4/PF-positive HEK-293 cell. The sequential depolarization of the taste cell by 70 mM KCl, as indicated by the line segments above the cAMP trace, elicited rather reproducible responsesof the serotonin biosensor. The deviations of cytosolic cAMP were quantified by the ratio $\Delta F/F_0$, where F = F-F_0, F is the instant fluorescence of the cAMP sensor Pink Flamindo, F_0 is the averaged fluorescence in the very beginning of the recording. The bath solution included (mM): 140 NaCl, 5 KCl, 1 $MgCl_2$, 2 $CaCl_2$, 10 HEPES-NaOH (pH 7.4), 10 glucose. The taste cell was stimulated by the substitution of 140 mM NaCl for 70 mM NaCl+70 mMKCl.

Note however that the enzymatic dissociation of taste buds, which was necessary to obtain individual taste cells, compromised their functionality. Since taste cells remained much more robust being embedded in a taste bud, stimulated serotonin secretion from

taste buds waspreferably studied at the level of individual taste buds, given that among taste bud cells, solely type III cells produced and released serotonin.

I. Effects of CASR ligands on ion currents in taste cells of the type II.

As a control for specificity, we studied effects of the CASR agonist NPS R-568 and antagonist NPS-2143 on VG currents in type II cells, which did not express CASR (Bystrova et al., 2010). Characteristic of these cells are VG Na^+ currents and outward currents via non-selective CALHMchannels. It turned out that NPS R-568 (0.5 µM) irreversibly diminished VG outward currents by33±5% (8 cells) compared to control (Fig.S3A, B) but affected VG Na^+ currents negligibly (Fig.S3,inset). Type II cells were weakly sensitive to 2 µM NPS-2143, which reversibly decreased VG Na^+ and outward currents by 6-14% (n=6) (Fig.S3C,D).

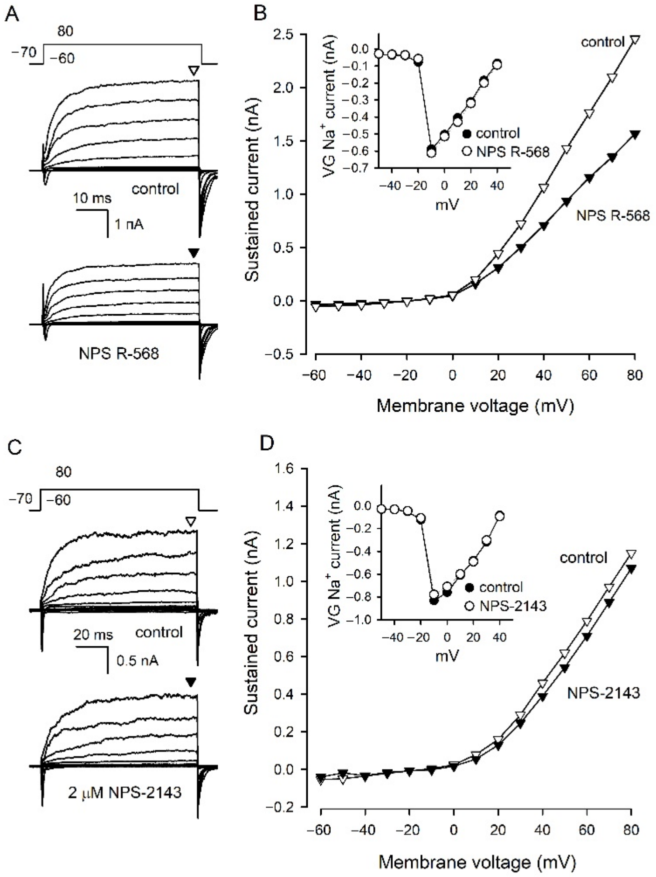

Figure S3. Effects of CASR ligands on VG currents in type II cells. (A) Representative (n=8) perforated patch recording of integral currents in control and in the presence of 0.5. µM NPS R-568. The cell was held at -70 mV and polarized from -60 to 80 mV by 50-ms voltage pulses with the 10-mV increment. Thepatch pipette contained 140 mM CsCl andAmphotericin B (400 µg/ml), the bath solutioncontained 140 mM NaCl. (B) Voltage dependence of the sustained current shown in (A) in control (∇) and in the presence of 0.5µM NPS R-568 (▼). The sustained currents were evaluated at the moments indicated by the symbols above the current traces. Insert, the I-V curve of the VG Na^+ currents shown in (A) (C, D) Representative (n=6) recordings ofintegral currents in control and in the presenceof 2 µM NPS-2143 and their I-V curves (D). Recording conditions as in (A).

I. Assay of serotonin release from foliate taste buds.

The abundance and robustness of individual type III cells in a preparation of dissociated taste budswere critical for accomplishing this study. The CV papilla as a taste cell source was more preferable than the foliate papilla because the much larger number of

sufficiently robust cells of the type III could be isolated from it. Nevertheless, foliate cells of the type III were also assayed in the limited number of experiments. As was found, foliate cells of the type III also exhibited theinvariancy of depolarization-induced release of serotonin at varied bath Ca^{2+} (Fig.S4).

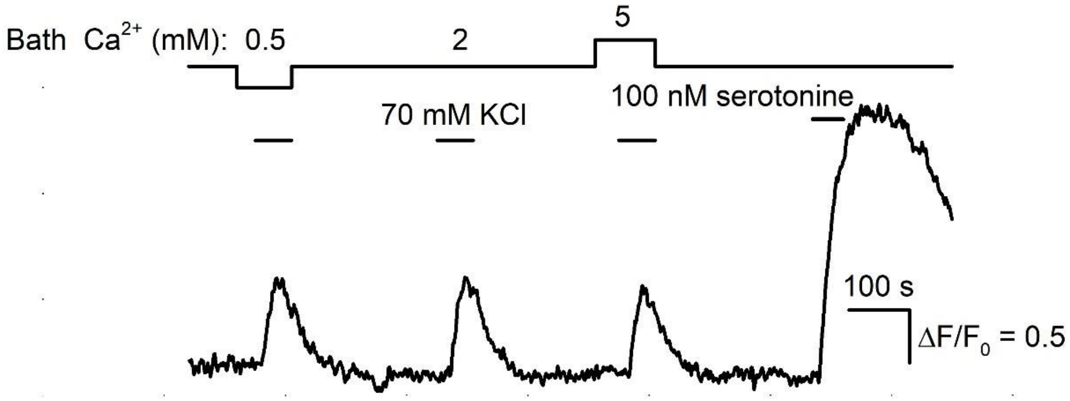

Figure S4. Assay of serotonin release from a foliate taste bud with the 5-HT4/PF- biosensor at varied bath Ca^{2+}. The representative (n-5) bottom trace represents the evolution of the cAMP signal in the serotonin biosensor during the assay. The depolarization of taste bud cells by 70 mM KCl stimulated serotonin release that wasapparently unaffected by a change in bath Ca^{2+} (solid line above the cAMP trace). The control application of exogeneous serotonin (100 nM) elicited the much higher response, indicating that the biosensor was not saturated by released serotonin. The deviations of cytosolic cAMP were quantified by the ratio $\Delta F/F_0$, where $\Delta F = F\text{-}F_0$, Fis the instant fluorescence of the cAMP sensor Pink Flamindo, F_0 is the averaged fluorescence in the very beginning of the recording.
